# Neoadjuvant chemoradiotherapy combined with immunotherapy for locally advanced rectal cancer: A new era for anal preservation

**DOI:** 10.3389/fimmu.2022.1067036

**Published:** 2022-12-08

**Authors:** Yaqi Wang, Lijun Shen, Juefeng Wan, Hui Zhang, Ruiyan Wu, Jingwen Wang, Yan Wang, Ye Xu, Sanjun Cai, Zhen Zhang, Fan Xia

**Affiliations:** ^1^ Department of Radiation Oncology, Fudan University Shanghai Cancer Center, Shanghai, China; ^2^ Department of Oncology, Shanghai Medical College, Fudan University, Shanghai, China; ^3^ Shanghai Clinical Research Center for Radiation Oncology, Shanghai, China; ^4^ Shanghai Key Laboratory of Radiation Oncology, Shanghai, China; ^5^ Department of Colorectal Surgery, Fudan University Shanghai Cancer Center, Shanghai, China

**Keywords:** locally advanced rectal cancer, neoadjuvant chemoradiotherapy, immunotherapy, tumor response, anus preservation, research progress

## Abstract

For locally advanced (T3-4/N+M0) rectal cancer (LARC), neoadjuvant chemoradiotherapy (nCRT) followed by total mesorectal excision (TME) is the standard treatment. It was demonstrated to decrease the local recurrence rate and increase the tumor response grade. However, the distant metastasis remains an unresolved issue. And the demand for anus preservation and better quality of life increases in recent years. Radiotherapy and immunotherapy can be supplement to each other and the combination of the two treatments has a good theoretical basis. Recently, multiple clinical trials are ongoing in terms of the combination of nCRT and immunotherapy in LARC. It was reported that these trials achieved promising short-term efficacy in both MSI-H and MSS rectal cancers, which could further improve the rate of clinical complete response (cCR) and pathological complete response (pCR), so that increase the possibility of ‘Watch and Wait (W&W)’ approach. However, the cCR and pCR is not always consistent, which occurs more frequent when nCRT is combined with immunotherapy. Thus, the efficacy evaluation after neoadjuvant therapy is an important issue for patient selection of W&W approach. Evaluating the cCR accurately needs the combination of multiple traditional examinations, new detective methods, such as PET-CT, ctDNA-MRD and various omics studies. And finding accurate biomarkers can help guide the risk stratification and treatment decisions. And large-scale clinical trials need to be performed in the future to demonstrate the surprising efficacy and to explore the long-term prognosis.

## Introduction

For locally advanced rectal cancer (LARC, T3-4/N+M0), the standard treatment is neoadjuvant chemoradiotherapy (nCRT) combined with total mesorectal excision (TME) followed by adjuvant chemotherapy. nCRT reduces local recurrence, promotes tumor downgrading, and improves the anus-preservation rate. Some patients can even achieve pathological complete response (pCR) (1), thus obtaining a better prognosis. Patients who achieve a clinical complete response (cCR) are also candidates for the nonoperative watch and wait (W&W) approach (2) to obtain better quality of life. However, given the increasing demand to achieve tumor regression and anus preservation in recent years, the traditional nCRT treatment model has encountered a bottleneck. Further improving tumor regression and long-term survival has become a challenge. In addition, reducing toxicity and improving quality of life also represent important issues. At present, significant efforts have been made to develop immunotherapy-based regimens. The use of nCRT combined with immunotherapy has also been studied recently in LARC patients, yielding gratifying short-term efficacy. The Immune-stimulating effect of nCRT could potentially overcome the resistance of microsatellite stable (MSS) colorectal cancer to immunotherapy and serve as a good paradigm for achieving synergistic optimization.

## Current status of neoadjuvant chemoradiotherapy for locally advanced rectal cancer

For LARC patients, neoadjuvant radiotherapy includes long-course radiotherapy (LCRT, 50 Gy/25 Fx, 5-Fu or capecitabine sensitization) and short-course radiotherapy (SCRT, 25 Gy/5 Fx). nCRT can significantly reduce the local recurrence rate (<10%) and has become the standard treatment based on NCCN guidelines. The pCR rate for standard treatment is only 10-20%, and it did not improve the long-term prognosis.

To improve the efficacy of neoadjuvant therapy, researchers have attempted to increase the treatment intensity of nCRT. The CinClare study confirmed that the addition of irinotecan to nCRT could increase the pCR rate compared with the standard nCRT group (30.0% vs 15.0%) (3). Recently, researchers have tried to advance adjuvant chemotherapy ahead of surgery through the use of consolidation chemotherapy (4), induction chemotherapy (5), or even total neoadjuvant therapy (TNT) (6). Multiple clinical trials demonstrated that TNT could increase the complete response (CR, pCR + cCR) rate to greater than 30% and improve the anus-preservation rate. TNT also improved compliance with nCRT and controlled distant metastasis early, providing patients with sufficient systemic treatment to achieve long-term benefits.

However, given the increasing demand for anal preservation and good quality of life, the CR rate of TNT remains insufficient, especially for those with tumors in lower locations. To further improve efficacy, risk stratification, population selection, sensitivity testing and precision treatment are new directions worth exploring. New antitumor methods, such as immunotherapy (PD-1/PD-L1 monoclonal antibody), have gradually been introduced to the field of nCRT for LARC patients.

## Theoretical basis of nCRT combined with immunotherapy

In recent years, immunotherapy has achieved great success in the treatment of a variety of malignant tumors and has become a new pillar of anticancer treatment. Microsatellite instability-high (MSI-H) patients have a higher tumor mutation burden (TMB) and increased tumor-infiltrating lymphocytes (TILs), which have naturally high sensitivity for immunotherapy (7, 8). The Keynote-177 study suggested that pembrolizumab monotherapy should be used as a new standard of first-line treatment for patients with metastatic dMMR/MSI-H colorectal cancer (9). However, dMMR/MSI-H tumors accounted for less than 5% of colorectal tumors, and greater than 95% of the tumors were classified as MSS tumors that are not very sensitive to immunotherapy alone. Therefore, improving the efficacy of MSS colorectal cancer is of great significance.

Preclinical studies have shown that radiotherapy promotes antitumor immunity. Radiotherapy induces the immunogenic death (ICD) of tumor cells; releases proinflammatory signals, such as neoantigens and damage-associated molecular patterns (DAMPs); and promotes the activation of antitumor T cells and the accumulation of TILs (10, 11). Radiotherapy can induce the upregulation of PD-L1 expression in tumor tissues and increase the sensitivity to immunotherapy. Radiotherapy combined with PD-L1 antibody can simultaneously regulate the tumor microenvironment, relieve its immunosuppressive effect, and enhance T-cell-derived antitumor cytokines (12, 13). Clinical studies of radiotherapy combined with immunotherapy have also observed the ‘abscopal effect’ (14, 15), which means that when a tumor at a certain location was treated with radiation, the tumor at another remote location also achieved a significant regression at the same time, probably because the radiation activates the systemic immune response. The above evidence shows that radiotherapy is expected to become one of the best types of therapy to combine with immunotherapy. Thus, through a mutual sensitization effect, the combination of nCRT and immunotherapy promotes synergism between local and systemic treatments, thereby achieving better tumor regression and long-term prognosis.

And the pathological files of the surgical specimens after nCRT and immunotherapy demonstrated the above changes, mainly including the following three features (16), i) Immune activation, as indicated by lymphoid infiltrates, tertiary lymphoid structures, and plasma cells; ii) Cell death, signified by foamy macrophages and cholesterol clefts; iii) The identification and histologic description of a tumor regression, including features of tissue repair, in particular proliferative fibrosis and neovascularization. And in terms of the LARC after nCRT plus immunotherapy in our cancer center, lymphocytic infiltration, cell necrosis and proliferative fibrosis and neovascularization are also found in post-surgery specimens.

Currently, the application of immunotherapy has gradually moved from posterior-line therapy to first-line therapy for metastatic cancers and has begun to be applied to adjuvant and neoadjuvant therapies for early-stage cancers. At the stage of neoadjuvant therapy, patients are generally in a better condition and are more susceptible to adverse treatment reactions. In terms of neoadjuvant therapy for colorectal cancer, the NICHE study and the PICC study confirmed that immunotherapy can increase the pCR rate of MSI-H/dMMR patients to greater than 60% (17, 18). At the 2022 ASCO Annual Meeting, it was reported that PD-1 monotherapy used in MSI-H/dMMR LARC achieved a cCR rate of 100% (14/14) (19). For MSS LARC patients, an increasing number of researchers are exploring the use of chemoradiotherapy combined with PD-1/PD-L1 inhibitors, and good results have been obtained.

## Clinical trials of nCRT combined with immunotherapy

Currently, numerous clinical studies on nCRT combined with immunotherapy have reported preliminary results. Most of the studies were prospective stage I-II trials with small sample sizes. The enrolled patients were mainly classified as having MSS tumors. The primary endpoints included the main indicators of tumor regression, such as the pCR rate, cCR rate, TRG, and NAR score, as well as treatment safety (the incidence of adverse reactions). The study designs included both LCRT (conventional fractionated radiation, 50 Gy/25 Fx) and SCRT (hypofractionated radiation, 25 Gy/5 Fx), and the sequences of immunotherapy and nCRT varied greatly (sequential or concurrent). Major information of clinical trials with published data is summarized in **Table 1** (LCRT-based trials) and **Table 2** (SCRT-based trials). And for detailed information of all ongoing clinical trials, please refer to **Supplementary Table 1** and **Supplementary Table 2**.

**Table 1:** Major clinical trials of the long course radiotherapy (LCRT) combined with immunotherapy for LARC.

Study	Phase	No.	Features	Study design	Results
Voltage-A	Ib	37	III 23%	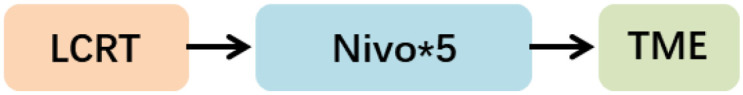	pCR 30%
NSABP FR-2	II	45	III 89%	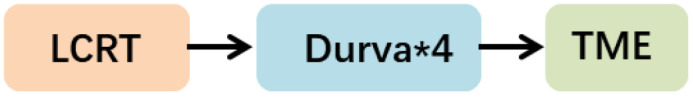	mNAR 12.03 pCR 22.2% cCR 31.1%
PANDORA	II	55	T3-4 95% N+ 79%	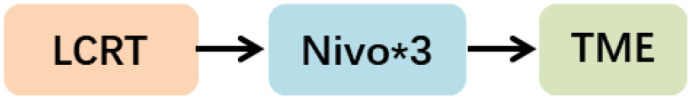	pCR 32.7% MPR 25.5%
AVANA	II	100	III 94%	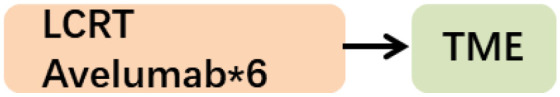	pCR 23% MPR 60%
R-IMMUNE	Ib	25	III 92%	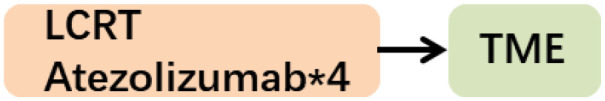	pCR 24%
NRG -GI002	II	95	High risk+	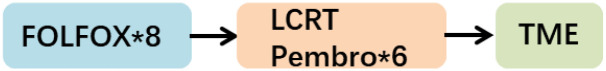	pCR 31.9% cCR 13.9%
PKUCH 04	II	25	76% N2 56% MRF+	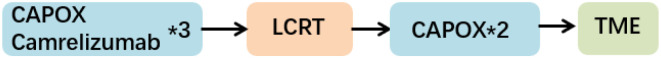	pCR 33.3% cCR 16% MPR 25%
Changhai	II	23	Super-low cT2 56.5% cN0 69.6%	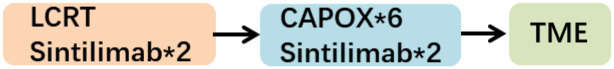	cCR 43.5% ncCR 26.1% Anal preservation 95.5% CR 52.2%
Beijing Friendship	II	12	cT3N0 cT1-3N+	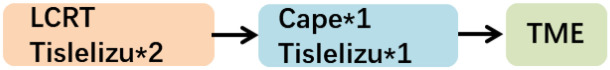	pCR 58.3%

**Table 2:** Major clinical trials of the short course radiotherapy (SCRT) combined with immunotherapy for LARC.

Study	Phase	No.	Features	Study design	Results
Wuhan	II	26	High risk+	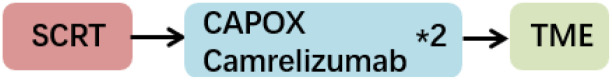	pCR 46%
Averectal	II	40	III 91%	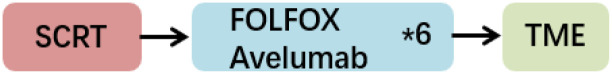	pCR 37.5% MPR 67.5%

### Long-course nCRT combined with immunotherapy

LCRT and immunotherapy are mainly combined through two modes: sequential or concurrent.

The trials using sequential immunotherapy followed by nCRT mainly included VOLTAGE-A, NSABPFR-2, and PANDORA, all of which achieved better tumor responses than the standard nCRT regimen. The first results were reported by the VOLTAGE-A study in Japan (20). This study used traditional nCRT (50.4 Gy/25 Fx/capecitabine) followed by 5 courses of nivolumab monotherapy. The results showed that among the 37 MSS patients, 11 patients achieved pCR (30%), 3 patients achieved near-pCR (8%), and 1 patient achieved cCR and adopted the W&W approach. Only 3 patients experienced grade 3-4 toxicities. The American NSABPFR-2 trial (21) reported at the 2022 ASCO GI conference enrolled 45 patients with stage II-IV rectal cancer who received 4 courses of durvalumab monotherapy after nCRT followed by TME surgery. The primary endpoint was the neoadjuvant therapy (NAR) score. The results showed that the patient’s mNAR score was 12.03, the pCR rate was 22.2%, the cCR rate was 31.1%, the R0 resection rate was 81.0%, and the anus-preservation rate was 71.4%. The main grade 3 toxicities included diarrhea, lymphopenia and low back pain. Only one patient had grade 4 adverse reactions (elevated amylase/lipase). Results from the Italian PANDORA study were reported at the 2022 ASCO Annual Meeting (22). The study used the Simon two-stage design. A total of 55 patients with LARC were enrolled. After nCRT (50.4 Gy/25 Fx/capecitabine), three courses of durvalumab monotherapy were administered. The results showed that 34.5% (19/55) of patients achieved pCR (TRG 0), 25.5% (14/55) of patients achieved near-pCR (TRG 1), and the Major Pathologic Response (MPR, less than 10% of the residual tumor compared with the baseline) rate was 60.0%. In addition, a low rate of grade 3-4 toxicities for nCRT or durvalumab was observed. The pCR rate of traditional nCRT was approximately 15-20%, and the overall CR rates (pCR + cCR) in these three studies all reached 30% or greater, suggesting that the combination of nCRT and immunotherapy achieved a good tumor response.

Trials of nCRT concurrent with immunotherapy included the ANAVA and R-IMMUE studies. The Italian ANAVA study reported at the 2021 ASCO Annual Meeting (23) enrolled 101 LARC patients. These patients were administered 6 courses of avelumab starting on the first day of nCRT. Among the 96 patients with final pathological results, 22 cases (23%) reached pCR, and 59 cases (61.5%) reached MPR. The rates of grade 3-4 nonimmune and immune-related toxicity were only 8% and 4%, respectively. The 2021 ESMO Annual Meeting reported the preliminary results of the Belgian R-IMMUNE study (24). Currently, the enrollment and treatment of phase Ib (6 cases) and the first stage (20 cases) of phase II (Simon two-stage design) have been completed. The enrolled patients were randomly divided into the standard nCRT group (45-50 Gy/5-Fu) and the nCRT-immunotherapy combination group (45-50 Gy/5-Fu + atezolizumab for 4 courses). The primary endpoint was the toxicity rate, and the secondary endpoint was the pCR rate. Preliminary results reported that 13% of patients experienced grade 3-4 toxicities (20/151 adverse events, including anastomotic fistula and infection in 10% of patients, urinary infection in 20%, renal function impairment in 5%, and immune-related thrombocytopenia in 5%; 34.6% (9/26) patients), and the pCR rate was 24% (6/25).

In addition, two additional studies implemented a TNT-like design. The 2021 ASCO-GI conference reported the results of the pembrolizumab cohort of the NRG-GI002 study (25). The control group received 8 courses of FOLFOX chemotherapy followed by nCRT concurrent with capecitabine, and the study group received 8 courses of FOLFOX chemotherapy followed by nCRT concurrent with capecitabine and pembrolizumab. The study endpoint was the NAR score. The results showed that the average NAR score of the control group and the pembrolizumab group were 14.08 and 11.53, respectively, and the difference was not statistically significant (P=0.26). The pCR values for the control group and the pembrolizumab group were 29.4% vs. 31.9% (P=0.75), respectively, and the cCR values were 13.6% vs. 13.9% (P=0.95), respectively. Although the statistical results showed that the tumor regression rates were similar in the two groups, the pCR+cCR rates in both groups were as high as approximately 44%. Thus, approximately half of patients achieved complete tumor remission, suggesting that the combination of the TNT pattern and immunotherapy is conducive to achieving the maximum degree of tumor regression. However, the addition of immunotherapy in this study failed to further improve tumor regression, which may be related to the low completion rate of pembrolizumab. Moreover, because lymphocytes are very sensitive to radiation, when radiotherapy is concurrently used with immunotherapy, radiotherapy may kill locally accumulated or activated lymphocytes, thereby adversely affecting the immune response.

Another PKUCH04 study conducted in Beijing (26) included 25 high-risk LARC patients. These patients received 3 courses of CAPOX chemotherapy combined with camerelizumab followed by LCRT, 2 courses of CAPOX chemotherapy and finally TME surgery or the W&W strategy. Eventually, 21 patients underwent TME surgery, 7 patients achieved pCR (33.3%, 7/21), and 15 patients achieved MPR (71.4%, 15/21). The remaining 4 patients achieved cCR or near-cCR after neoadjuvant therapy and eventually chose the W&W strategy. The major grade 3-4 adverse reactions included lymphopenia in 24% of patients, diarrhea in 8%, and platelet reduction in 4%. No grade 4 adverse reactions occurred. The above two TNT-designed nCRT combined with immunotherapy achieved a CR rate of approximately 50% in LARC patients, significantly improving tumor regression compared with the traditional nCRT model.

### Short-course radiotherapy combined with immunotherapy

SCRT and sequential chemotherapy are also commonly used modes of neoadjuvant treatment for LARC patients that can achieve pCR rates similar to those of LCRT. Studies have shown that the combination of hypofractionated SCRT and immunotherapy has more advantages. Hypofractionated SCRT has less of an effect on the peripheral blood lymphocytes of patients, thus promoting the antitumor effect of the immune system (27). Hypofractionated SCRT inhibits the recruitment of myeloid-derived suppressor cells (MDSCs) into tumors, reduces the expression of PD-L1 on the tumor surface and achieves a tumor growth inhibition rate that is superior to that of conventional fractionation (28). Moreover, abscopal effects in mice were also observed when hypofractionated radiotherapy was combined with immunotherapy (29).

A Chinese phase II study assessed SCRT (25 Gy/5 Fx) followed by 2 courses of XELOX plus carrelizumab and TME surgery (30). Among the 27 patients who underwent surgery (26 pMMR patients and 1 dMMR patient), the pCR rate was as high as 48% (13/27). The pCR rate in the pMMR subgroup was 46% (12/26), and the pCR rate in the dMMR subgroup was 100% (1/1). The R0 resection rate was 100%, the anal preservation rate was 89% (24/27), and the tumor downstaging rate was 70% (19/27). In this study, most patients had high risk factors for recurrence and metastasis (T4/N2/MRF+). This study achieved a very high pCR rate within only 2 months. This value even exceeded the pCR rate of the TNT model, which had the highest treatment intensity. These findings suggest that the combination of hypofractionated radiotherapy and immunotherapy may have a better effect, which was consistent with preclinical research. In addition, no serious adverse effects were observed, and grade 3 hematological toxicity could be alleviated after treatment in time. At present, a phase III multicenter clinical study is ongoing in the same cancer center.

The Averectal study conducted in Lebanon and Jordan recruited a total of 44 LARC patients who were treated with SCRT combined with 6 courses of mFOLFOX6 and avelumab (31). Except for 4 patients who were excluded from the analysis for various reasons, 15 of the remaining 40 patients achieved pCR (37.5%), and 12 patients achieved near-pCR (TRG 1, 30%). Thus, 67.5% of patients achieved very significant tumor regression. Additionally, the patients did not experience grade 3-4 immune-related toxicity, and the incidence of grade 3-4 surgery-related complications was only 5%. In addition, immunohistochemical staining of TILs was performed to calculate the immunoscore (IS). It was found that a higher IS was associated with a higher pCR rate.

In addition, another phase II trial named the TORCH study adopted the SCRT-based TNT model (32, 33) and reported surprising efficacy results for tumor regression at the 2022 ASCO Annual Meeting. This study included 130 patients who were randomly divided into the consolidation group and the induction group. Patients in the consolidation group first received SCRT followed by 6 courses of CAPOX and toripalimab. Patients in the induction group received 2 courses of CAPOX and toripalimab followed by SCRT and then 4 courses of CAPOX and toripalimab. Finally, the patients achieving cCR underwent TME surgery or adopted the W&W strategy. To date, 48 patients have completed neoadjuvant therapy. Twenty-four (24/48, 50.0%) achieved cCR, and 12 of them adopted the W&W strategy. Twenty-nine patients underwent TME surgery. The CR rate was 60.4% (29/48), the pCR rate was 60.7% (17/28), the MPR rate was 78.6% (22/28), and the anus-preservation rate was 88.9% (40/45). However, the follow-up period for W&W patients in the TORCH study was still relatively short, and the above data should be further updated after large sample size recruitment and long-term follow-up.

### Radiotherapy combined with immunotherapy for early rectal cancer

Traditionally, direct surgery is recommended as the standard treatment for early-stage rectal cancer. However, in recent years, the need for organ function preservation has increased considerably. Early-stage tumors could achieve a higher CR rate through neoadjuvant therapy. Thus, neoadjuvant therapy has important clinical significance in patients with early-stage low rectal cancer. The 2022 ASCO Annual Meeting reported a phase II study from China (34), which enrolled 23 patients with T1-3aN0-1 ultralow rectal cancer who underwent 2 courses of sintilimab during the same period of LCRT followed by 6 courses of sintilimab combined with capecitabine or CAPOX. Among the included patients, the baseline T2 rate was 56.5%, and the N0 rate was 69.6% (16/23). The cCR rate was 43.2% (10/23), the pCR rate was 20% (2/10), the CR (cCR + pCR) rate was 52.2% (12/23), and the anus-preservation rate was as high as 95.5% (21/22). The grade 3-4 toxicity rate was 17.4%. This study suggests that for early and mid-stage rectal cancer patients in whom anus preservation is difficult, nCRT combined with immunotherapy is expected to become a treatment option with high efficacy and low toxicity.

## Opportunities and challenges of neoadjuvant chemoradiotherapy combined with immunotherapy

The combination of nCRT and immunotherapy in LARC has achieved an excellent pCR/cCR rate and an increased anal preservation rate, which indicates that a new era for anal preservation is coming. However, there are still many challenges remaining to be resolved, including the accuracy of efficacy evaluation, the sequences of radiation and chemotherapy/immunotherapy, biomarker analyses and the survival benefit, etc.

### The new era of nCRT combined with immunotherapy

For LARC patients, the initial purpose of neoadjuvant chemoradiotherapy is to reduce local recurrence. However, the pCR rate is low, and distant metastasis has become the main failure mode of treatment. With the gradual optimization of neoadjuvant chemoradiotherapy regimens (such as enhancing the intensity of concurrent chemotherapy, increasing the cycles of interval chemotherapy, and even performing total neoadjuvant treatment (TNT)), the efficacy of tumor regression in patients gradually improved. Studies have shown that the TNT model can significantly increase the pCR rate to greater than 30%. More patients with cCR can adopt the nonsurgical W&W strategy, which increases the organ preservation rate and improves the quality of life. In addition, this strategy is expected to reduce distant metastasis and improve long-term survival. Therefore, neoadjuvant therapy for LARC has been transformed from the traditional era with the main purpose of controlling local recurrence to the new era with the goal of improving tumor regression, organ preservation, and long-term survival. The addition of immunotherapy has led to a more promising results in the new era. Although the results currently reported are mainly phase II small-sample studies, the results of studies with similar designs have good consistency.

In terms of LCRT-based clinical trials, except for the Voltage-A study (stage III 23%) and the Changhai study (ultralow cN0 69.6%), the vast majority of studies included greater than 85% of stage III patients. Most of the patients contained at least one feature of high risk of recurrence and metastasis (cN2/MRF+/EMVI+). In these patients, the CR rate of LCRT combined with immunotherapy can reach more than 30% (Voltage-A, NSABP FR-2, PANDORA). Thus, a CR rate similar to that of the TNT model can be achieved by only combining LCRT with PD-1 monoclonal antibody. On this basis, the combined immunotherapy-TNT model (NRG-GI002, PKUCH04, Changhai Hospital study) achieved a higher CR rate, and the pCR + cCR rates of the three studies were all 50% or greater. These findings represent a solid foundation for low rectal cancer patients to adopt the W&W strategy or reduce the scope of surgery to achieve organ preservation.

In terms of SCRT combined with immunotherapy, although a few clinical trials have published results, they all showed surprisingly high tumor regression efficacy with a pCR rate of 37.5-57.1%. The main model was SCRT followed by several cycles of chemotherapy and immunotherapy. The Wuhan study only performed two sequential cycles of CAPOX and carrelizumab and achieved an ultrahigh efficacy of 46%. The FUSCC TORCH study adopted the TNT model and performed SCRT combined with 6 cycles of CAPOX and toripalimab. The pCR rate is currently as high as 60.7%, and the CR rate is 60.4%. The above results show that hypofractionated radiotherapy combined with immunotherapy has a more powerful synergistic effect.

### The challenge of evaluating the efficacy of nCRT combined with immunotherapy

Organ preservation has become one of the main goals of neoadjuvant therapy for rectal cancer, especially in patients with low rectal cancer. For patients with cCR after nCRT, the W&W strategy can be performed under close follow-up. If tumor regression is good, local resection of the lesion can also be performed through the anus, and the decision of supplementary TME is based on postoperative pathological characteristics. If significant residual tumor remains, radical surgery (TME) is recommended as soon as possible. Therefore, the evaluation of the efficacy of neoadjuvant therapy is very important and is an important basis for the selection of subsequent treatment strategies.

Currently, the main methods for assessing clinical response (including cCR) include digital anal examination (DRE), colonoscopy (for lesions or suspicious lesions with biopsy), pelvic MRI, transrectal ultrasonography (TRUS), and serum tumor marker levels (such as CEA). And the evaluation methods and criteria across different centers are not consistent (**Table 3**). Each of them has its strengths and weaknesses. Thus, the efficacy evaluation needs the combination of the above methods. However, the consistency between the existing methods for the judgment of cCR after nCRT is not high with pCR. Smith FM et al. reported that pathological findings of 27% of cCR patients showed residual tumor cells (35). A considerable proportion of the rectal cancer tissue specimens with pCR did not meet the cCR criteria, mainly manifested as mucosal ulcer changes. The addition of immunotherapy may further increase the difference between imaging and pathological assessment results. For patients with effective immunotherapy, due to the increase in immune cell infiltration in the lesion, imaging studies may show that the lesion is stable or even enlarged, indicating false progression. Studies have shown that 14.8% of patients with metastatic CRC treated with PD-1 inhibitors had pseudoprogression (36). The presence of mixed signals of edema and fibrosis in the original tumor area after nCRT for rectal cancer may also interfere with the MR assessment of residual tumor. In addition, the uncertainty of imaging in the efficacy evaluation of immunotherapy also partially explains the small effect of the iRECIST criteria on the survival endpoint (37).

**Table 3:** The different evaluation methods across different cancer centers.

Institutions	Country	Year	Evaluation Methods
Sao Paulo Hospital	Brazil	2014	DRE, Endoscopy, MRI, TRUS, PET-CT
Maastricht University Medical Center	Netherlands	2011	Endoscopy and biopsy, MRI
The OnCoRe Group	UK	2016	DRE, Endoscopy, MRI
MSKCC	USA	2015	DRE, Endoscopy, MRI
ESMO	Europe	2017	DRE, Endoscopy and biopsy, MRI, TRUS, CEA

The preliminary results of studies on nCRT combined with immunotherapy for LARC showed that the CR rate was significantly increased. However, the transformation of this large tumor response improvement to the benefit of organ preservation depends on accurate evaluation methods and predictive indicators. In addition to traditional imaging methods, detection methods with higher sensitivity, such as PET/CT and ctDNA-MRD, and multidimensional assessments, such as radiomics, are urgently needed. The combination and optimization of traditional methods and new assessments will improve the judgment of tumor regression and facilitate the selection of subsequent treatment strategies.

### The best sequence between radiation and chemotherapy/immunotherapy remains uncertain

The different sequences between radiation and chemotherapy/immunotherapy may lead to different effects. The use of radiation firstly can lead to the immunogenic cell death of tumor, release new tumor-associated antigens, promote the antigen-presenting function of dendritic cells and increase the infiltration of T lymphocytes, so radiation can promote the effect of sequential chemotherapy/immunotherapy. And the inductive use of chemotherapy/immunotherapy can also change the tumor microenvironment, promote the tumor angiogenesis and increase the oxygenic distribution, so that having a synergic effect with radiation.

The best sequence between radiation and chemotherapy/immunotherapy is uncertain. In terms of chemotherapy, both CAO/ARO/AIO-12 trial and OPRA trial compared the efficacy of inductive chemotherapy (chemotherapy before CRT) and consolidative chemotherapy (chemotherapy after CRT) and found that consolidative chemotherapy could obtain better pCR/cCR rates, so that leading to a higher anal preservation rate. In terms of the immunotherapy, animal experiments showed that radiation with concurrent immunotherapy achieved better tumor regression than radiation with sequential immunotherapy. And there is only one study (TORCH) conducted by China comparing the efficacy of induction immunotherapy and consolidative immunotherapy, which is recruiting now and didn’t have the exact results of subgroup analyses yet. Furthermore, the tumor microenvironment analyses of biopsy tissues after treating with different sequences are needed to examine the pathological changes and investigate the underlying mechanisms.

### Biomarker analysis helps screen for patients who will benefit from treatment

dMMR and MSI-H are recognized markers for predicting the efficacy of PD-1 inhibitors. However, greater than 95% of patients with rectal cancer have the pMMR/MSS type, which is not very sensitive to PD-1 inhibitors alone. Therefore, screening the population that may benefit from nCRT combined with immunotherapy is one of the key factors to improve efficacy. A small sample study from MSKCC found that the number of TILs was correlated with the efficacy of nCRT, suggesting that TILs may be involved in the tumor killing effect caused by nCRT (38). In a study conducted by Professor Galon’s team in France, 249 rectal cancer biopsy specimens were analyzed. The quantitative IS of CD8+ T lymphocytes in the tumor center and infiltration margin was correlated with the efficacy and prognosis of nCRT. None of the patients with high scores experienced tumor recurrence in the long-term follow-up, suggesting that this strategy can help to screen the population suitable for the W&W strategy (39).

Clinical trials of nCT combined with PD-1 inhibitors for LARC have also conducted biomarker analysis, including Voltage-A, NSABP FR-2, Averectal study, and Wuhan study. Voltage-A researchers used pretreatment tumor biopsy specimens for analysis. The results showed that the patients with greater than 1% of PD-L1-positive cells in the tumor microenvironment and the patients with CD8/eTreg>2.5 had a higher pCR rate. The 5 patients with both of the above features all achieved pCR, suggesting that the number and function of lymphocytes in the immune microenvironment were closely related to the efficacy of nCRT combined with immunotherapy. The Averectal study also analyzed baseline biopsy specimens and found that patients with high IS achieved a higher pCR rate. The Wuhan study showed that PD-L1 CPS ≥ 1 and TMB ≥ 10 were associated with a higher pCR rate.

In addition to commonly used immunotherapy-related biomarkers, such as MSI-H, PD-L1, immune score (or TIL quantitative analysis), and TMB, mutations in specific genes or pathways (such as POLE or POLD1 mutations, B2M or JAK1/2 mutations), the molecular structure and phenotype of various proteins in the microenvironment, the types and quantitative characteristics of metabolites, and the composition and function of the intestinal microbial population may all affect the immune response and the efficacy of immunotherapy. With the broadening of the concept of the immune microenvironment and the further refinement of various omics studies, future studies will focus more on the establishment of relevant models that accurately predict efficacy and tumor recurrence and metastasis and use biomarkers to guide patient stratification and treatment decisions.

### The survival benefits of neoadjuvant immunotherapy remain to be confirmed

Regarding neoadjuvant therapy for LARC patients, in addition to the pCR rate/CR rate/organ preservation rate, recurrence-free survival (LRFS), disease progression-free survival (DFS), and overall survival (OS) are also important research endpoints. The existing nCRT model has greatly reduced the local recurrence rate to less than 10%. To control metastasis early and improve long-term survival, researchers have tried to move adjuvant chemotherapy forward and even perform the TNT model. The PRODIGE 23 study (40, 41) showed that compared with standard nCRT, 6 cycles of FOLFIRINOX chemotherapy before nCRT not only further increased the pCR rate (28% vs. 12%) but also significantly increased 3-y DFS% (76% vs. 69%) and 3-y DMFS% (79% vs. 72%). The RAPIDO study (42) showed that compared with standard nCRT, sequential 6 cycles of CAPOX or 9 cycles of FOLFOX chemotherapy after SCRT not only increased the pCR rate (28% vs. 14%) but also significantly reduced the disease-related treatment failure rate (DrTF, 23.7% vs. 30.4%) and distant metastasis rate (20.0% vs. 26.8%). The above results show that strengthening the intensity of neoadjuvant treatment and adopting the TNT model further promote tumor regression and have the potential to reduce metastasis and prolong long-term survival. The combination of nCRT and immunotherapy allows patients to receive systemic treatment earlier, which is expected to kill minimal disease earlier and reduce distant metastasis. Neoadjuvant immunotherapy can help the body obtain long-term immune memory by promoting the immune response and probably producing the smearing effect, finally achieving sustained tumor remission and long-term survival benefits. The above clinical trials have shown good short-term efficacy, but the follow-up time is insufficient. The tumor regression, toxicity and long-term survival caused by nCRT combined with immunotherapy deserve to be verified by phase III large-sample randomized controlled trials as soon as possible.

## Summary

In the neoadjuvant treatment of LARC, the new model of nCRT combined with immunotherapy has shown good application potential and is expected to break through the bottleneck of the limited cCR/pCR rate of traditional nCRT and the low response rate of immunotherapy for MSS rectal cancer. An increasing number of studies have reported that radiotherapy combined with immunotherapy can significantly improve tumor regression and the CR rate and is safe and tolerable, thereby providing low rectal cancer patients with more opportunities to adopt the W&W strategy. In the future, we need more large-scale clinical trials to validate this new model and to explore how to improve the efficacy evaluation, select populations that would benefit based on various biomarkers, and explore the optimization model of the combination therapy. We look forward to converting the surprising short-term efficacy into improved survival time and quality of life for LARC patients.

## Author contributions

FX selected the topic. Ji W, RW, and Yan W searched the registered clinical trials and prepared the **Supplementary Tables**. Yaq W reviewed all published articles and posters, and wrote the manuscript. Ju W and ZZ provided some revising suggestions on the Discussion section. JW, LS, HZ, and SC revised the manuscript. All of the authors critically read and approved the manuscript.

## Supplementary material

Supplementary Table 1 The clinical trials of the long course radiotherapy combined with immunotherapy.

Supplementary Table 2 The clinical trials of the short course radiotherapy combined with immunotherapy.
